# Treatment of COVID-19 With Conestat Alfa, a Regulator of the Complement, Contact Activation and Kallikrein-Kinin System

**DOI:** 10.3389/fimmu.2020.02072

**Published:** 2020-08-14

**Authors:** Pascal Urwyler, Stephan Moser, Panteleimon Charitos, Ingmar A. F. M. Heijnen, Melanie Rudin, Gregor Sommer, Bruno M. Giannetti, Stefano Bassetti, Parham Sendi, Marten Trendelenburg, Michael Osthoff

**Affiliations:** ^1^Division of Internal Medicine, University Hospital Basel, Basel, Switzerland; ^2^Laboratory Medicine, Division of Medical Immunology, University Hospital Basel, Basel, Switzerland; ^3^Clinic of Radiology and Nuclear Medicine, University Hospital Basel, Basel, Switzerland; ^4^Pharming Group, Leiden, Netherlands; ^5^Department of Clinical Research and Department of Biomedicine, University Hospital Basel, Basel, Switzerland; ^6^Department of Infectious Diseases and Hospital Epidemiology, University Hospital Basel, Basel, Switzerland

**Keywords:** COVID-19, C1 esterase inhibitor, SARS-CoV-2, inflammation, complement system, kallikrein-kinin system, contact activation system

## Abstract

A dysregulated immune response with hyperinflammation is observed in patients with severe coronavirus disease 2019 (COVID-19). The aim of the present study was to assess the safety and potential benefits of human recombinant C1 esterase inhibitor (conestat alfa), a complement, contact activation and kallikrein-kinin system regulator, in severe COVID-19. Patients with evidence of progressive disease after 24 h including an oxygen saturation <93% at rest in ambient air were included at the University Hospital Basel, Switzerland in April 2020. Conestat alfa was administered by intravenous injections of 8400 IU followed by 3 additional doses of 4200 IU in 12-h intervals. Five patients (age range, 53–85 years; one woman) with severe COVID-19 pneumonia (11–39% lung involvement on computed tomography scan of the chest) were treated a median of 1 day (range 1–7 days) after admission. Treatment was well-tolerated. Immediate defervescence occurred, and inflammatory markers and oxygen supplementation decreased or stabilized in 4 patients (e.g., median C-reactive protein 203 (range 31–235) mg/L before vs. 32 (12–72) mg/L on day 5). Only one patient required mechanical ventilation. All patients recovered. C1INH concentrations were elevated before conestat alfa treatment. Levels of complement activation products declined after treatment. Viral loads in nasopharyngeal swabs declined in 4 patients. In this uncontrolled case series, targeting multiple inflammatory cascades by conestat alfa was safe and associated with clinical improvements in the majority of severe COVID-19 patients. Controlled clinical trials are needed to assess its safety and efficacy in preventing disease progression.

## Introduction

The current pandemia of coronavirus disease 2019 (COVID-19) caused by severe acute respiratory syndrome coronavirus 2 (SARS-CoV-2) remains a major global health challenge. The clinical spectrum of COVID-19 ranges from asymptomatic carriers to respiratory failure. A dysregulated immune response with evidence of hyperinflammation is observed in patients with severe COVID-19 (1). Siddiqi et al. suggested a disease stage model, where early disease is charaterized by infection and replication of the virus. Subsequently, pulmonary involvement and marked hyperinflammation occur during the next stage (2). The exact factors promoting disease progression are not yet known. However, sustained activation of the complement system (CS) induced by coronaviruses (CoV) seems to play a crucial role in this context.

The complement system (CS) is an integral part of the innate immune system and consists of a number of distinct plasma proteins that act as a first line of defense inducing an inflammatory response after opsonisation of pathogens and dying cells (3, 4). Inflammatory responses include the activation of macrophages, neutrophils, platelets and endothelial cells, and interactions with other plasmatic cascades. While the CS does not seem to be critical for controlling CoV replication, unregulated complement activation - induced by viruses including influenza and CoV - plays a crucial role in the pathogenesis of acute lung injury (ALI). Indeed, an animal model suggests that the CS mediates SARS-CoV-induced lung disease and regulates the proinflammatory response. Complement deficient mice infected with SARS-CoV were affected less severely and showed a reduced lung involvement and lower local and systemic cytokine levels compared to control mice (5). In line, inhibition of complement C5a signaling alleviated lung damage in animal infection models using MERS-CoV (6) and influenza H7N9 (7). Similar results were reported with inhibition of complement cascade C3a in an animal infection model using avian influenza (8). Gao et al. investigated the interaction of MERS-CoV, SARS-CoV, and SARS-CoV-2 with the lectin pathway of complement in more detail (9). They demonstrate an interaction of these highly pathogenic CoV with mannose-binding lectin associated serine protease-2 (MASP-2), the key activator of the lectin pathway of complement (10), leading to uncontrolled activation of the complement cascade. In line, MASP-2 knock-out mice and mice treated with MASP-2 inhibitors showed significantly milder symptoms in a virus protein mouse pneumonia model. Mannose-binding lectin, the pattern recognition molecule of the lectin pathway, that activates MASP-2 upon binding to pathogens, was found to bind to SARS-CoV spike glycoprotein (11). In COVID-19 patients, increased plasma levels of complement activation products C5a and soluble C5b-9 have been observed (12). Furthermore, autopsy findings from 5 patients with severe COVID-19 revealed excessive complement activation in lung tissue associated with complement-mediated microthrombotic disease (13).

Beside CS activation, involvement of the contact activation (CAS) and kallikrein-kinin system (KKS) in thromboinflammation, capillary leakage and subsequent pulmonary angioedema has been suspected. Angiotensin-converting enzyme 2 (ACE2), a cell membrane bound protein used by SARS-CoV-2 to enter cells, also inactivates bradykinin degradation products, and expression of ACE2 as well as its enzymatic activity is decreased by SARS-CoV (14). Hence, the interaction of SARS-CoV-2 with ACE2 may impair its function, leading to a relative abundance of bradykinin degradation products and local pulmonary edema.

The role of the CAS has not been elucidated in COVID-19. However, dysregulated coagulation is commonly observed in critically-ill patients with COVID-19 and thromboembolism has been reported more frequently compared to other diseases causing severe sepsis (15, 16). This may be a consequence of over-activation of CAS since sepsis and ARDS are prototypic states that strongly activate the CAS (17, 18). Consistent with thromboinflammation, microthrombi have been observed in autopsy series in COVID-19 (19).

C1 esterase inhibitor (C1INH) is a strong inhibitor of the CS, CAS, and KKS and interacts with stressed endothelial cells. C1INH treatment was associated with reduced capillary leakage after stem cell transplantation and organ damage in human sepsis (20–22). Recently, C1INH was identified among other inflammatory proteins to be upregulated in severe vs. non-severe COVID-19 in a proteomic and metabolomic characterization (23). In a mouse model of highly pathogenic CoV, C1INH treatment was able to block MASP-2 mediated overactivation of the complement system and subsequent lung injury and death (9). Lastly, when examining published interactomes of SARS-CoV-1 and−2, a significant interaction of C1INH with CoV-1 proteins was documented including proteins that are highly similar to their orthologous CoV-2 proteins (24).

Investigating a regulator of the CS, CAS, and KKS for the first time in severe COVID-19, we report the experience of early administration of conestat alfa, a recombinant human C1INH, in non-critically ill patients to prevent deterioration.

## Materials and Methods

The treatments occurred at the University Hospital Basel from April 2, 2020, to April 28, 2020 and were approved by the Ethics Committee of Northwest and Central Switzerland (EKNZ 2020-1013). All participants consented to the compassionate use of conestat alfa and the acquisition of health-related personal data and biological material.

### Study Drug

Conestat alfa is a recombinant human C1INH that shares an identical protein structure with plasma-derived C1INH but has a different glycosylation pattern. Comparable inhibition of most target proteases has been demonstrated (25). Despite the broad interference with several cascades and targets, major adverse events or unique toxicities have not been demonstrated in previous studies with the exception of a potential risk of allergic reactions in patients with rabbit dander allergy.

### Patients and Intervention

Patients with polymerase chain reaction-confirmed and severe COVID-19 (26) were included if they were at least 18 years old, showed evidence of progressive disease after 24 h, and had a C-reactive protein level of at least 30 mg/L and oxygen saturation of <93% at rest in ambient air. Exclusion criteria included admission to ICU, immunosuppression, and known allergy to rabbit dander. Conestat alfa was administered by 4 intravenous injections in 12-h intervals over 48 h. The dosage was identical for all patients irrespective of body weight (8400 IU as initial dosage followed by 4200 IU).

### Data Collection

Clinical information was collected from the electronic hospital information system. Affected lung tissue on a computed tomography (CT) scan was quantified semi-automatically using software for lung density analysis in Chest CT available at our radiology department [CT Pulmo 3D included in Syngo.Via VB30A, Siemens Healthineers, Forchheim, Germany; method similar as described recently (27)]. The processing included semi-automatic segmentation of the lungs followed by a threshold-analysis of Hounsfield units (HU), where pulmonary involvement was defined as the percentage of lung parenchyma with a CT-density between −600 and 0 HU. Concentration of complement proteins and C1INH antigen were analyzed in serum or plasma by standardized nephelometric assays (C3, C4, C1INH) or ELISA assays in duplicate according to the instructions of the manufacturer (C4d, C5a; Quidel, San Diego, U.S.A.). A control group was established from the database of all COVID-19 patients admitted during the same period (March and April 2020) at the University Hospital Basel (*n* = 165). Propensity score-matching (1:3 matching of cases and controls) was performed to adjust for relevant confounders (nearest-neighborhood method; caliper width of 0.25 times the standard deviation of the logit of propensity scores). The following variables were included in the matching model: gender (exact), age, lung involvement on CT scan of the chest and Charlson Comorbidity index. The maximum standardized difference of the mean was 0.05 and the area under the curve was 0.85. Characteristics of the group were compared with the use of the Fisher's exact test for categorical variables and the Mann-Whitney *U*-test for continuous variables.

## Results

**Figure 1:**
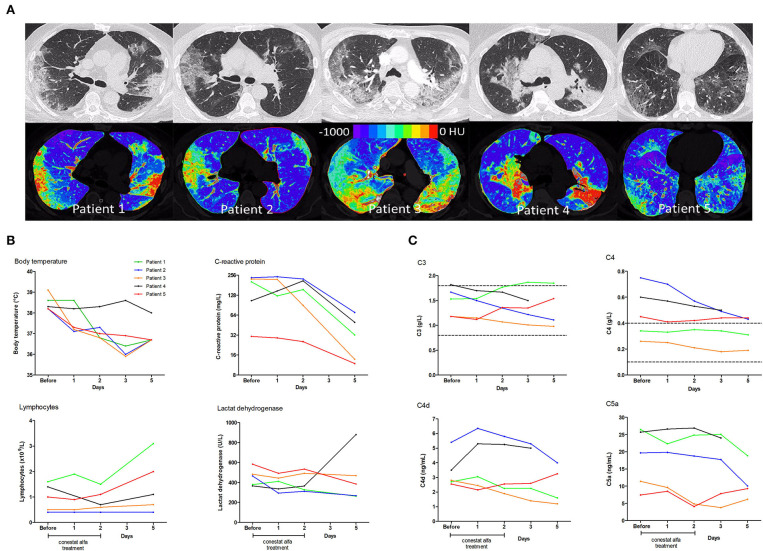
Chest computed tomography scans and temporal changes of laboratory parameters in five patients treated with conestat alfa. **(A)** Upper row: Original images in lung kernel reconstruction showing ground-glass opacities and consolidations involving multiple segments of both lungs with subpleural predominance. Lower row: color-coded maps from lung density analysis used to quantify the percentage of affected lung volume. **(B)** Temporal changes of body temperature, plasma C-reactive protein levels, lymphocyte counts and lactate dehydrogenase levels. **(C)** Temporal changes in concentration of serum complement component C3 and C4, and plasma activation products complement component C4d and C5a. Reference ranges are depicted by dashed lines.

Five patients (4 males, median age 60 years, range 53–85 years) were treated with conestat alfa. Four had preexisting medical conditions (Table 1). A CT scan of the chest demonstrated moderate to severe pneumonia with involvement of lung parenchyma ranging from 11 to 39% (Figure 1A). All patients received hydroxychloroquine and lopinavir/ritonavir (LPV/r) as per local treatment guideline at the time. Conestat alfa was administered a median of 1 day (range 1–7 days) after admission for 48 h and was well-tolerated. No treatment associated serious adverse events were recorded.

**Table 1:** Clinical characteristics of SARS-CoV-2 infected patients treated with conestat alfa.

**Characteristics^**a**^**	**Patient 1**	**Patient 2**	**Patient 3**	**Patient 4**	**Patient 5**
BMI	27.7	28.4	22.4	31.2	22.6
Smoking (ongoing or recent)	No	Yes	Yes	No	Yes
Comorbidity	CKD, hypertension, hypercholesterolemia, gout	CKD, CVD, diabetes, hypertension, PAD	CVD, hypertension	Asthma, hypertension, hypothyroidism	None
Days from symptom onset to admission	15	4	4	6	7
Days from admission to conestat alfa	1	1	7	1	2
Symptoms	Fever, diarrhea, fatigue, cough, chest pain	Cough, sore throat	Fatigue, diarrhea, muscle ache	Cough, diarrhea, fatigue, muscle ache, dyspnea	Fever, cough, fatigue, dyspnea
Lung involvement, %^b^	14	18	39	24	11
SOFA score day 0	1	2	2	1	2
NEWS2 score day 0	5	5	9	8	7
SARS-CoV-2 viral load day 0, copies/mL	23,500	36,046,600	1,000	611,900	33,400
Respiratory rate day 0, per minute	21	25	22	22	24
CRP day 0, mg/L	203	235	223	106	31
Ferritin day 0, μg/L	1,280	567	3,736	560	1,805
LDH day 0, U/L	379	466	483	366	584
D-dimer day 0, μg/ml	1.2	4.2	1.0	0.6	1.7
IL-6 day 0, ng/L	60	187	141	55	32
C1INH d0, g/l	0.71	0.45	0.57	0.52	0.64
C1INH d1, g/l	0.71	0.48	0.58	0.53	0.63

*CKD, chronic kidney disease; CVD, cardiovascular disease; PAD, peripheral artery disease; BMI, body mass index; SOFA, sepsis-related organ failure assessment score; NEWS2, National Early Warning Score 2; CRP, C-reactive protein; LDH, lactate dehydrogenase; IL-6, interleukin-6*.

a *Day 0 denotes the day of first administration*.

b *Lung involvement was determined from computed tomography scans of the chest*.

### Outcome

Immediate defervescence occurred in all but one patient (patient 4) within 48 h (Figure 1B). Three patients were weaned off oxygen supplementation and discharged early. In patient 3, conestat alfa was administered late during admission (day 7) after rapid progression (pulmonary involvement increased from 7 to 39%). Hence, only 12 h after the start of conestat alfa tocilizumab was also administered. Only 1 patient deteriorated after 48 h of treatment and required mechanical ventilation. Tocilizumab and amoxicillin/clavulanic acid were administered. The patient was extubated after 10 days. All patients were discharged within 3 weeks (range 6–20 days).

Outcome was compared to a matched control population of 15 patients. Baseline characteristics, admission laboratory parameters and treatments administered were similar in both groups (Table 2), except for present smoking [3/5 (60%) of the conestat alfa group and 0/15 (0%) for the matched control population]. However, 8/15 (53%) patients in the control population required mechanical ventilation or died, compared to only 1 (20%) in the conestat alfa group.

**Table 2:** Comparison of demographics, clinical features and outcome of 5 SARS-CoV-2 infected patients treated with conestat alfa and a matched control group.

**Variable**	**Control group *N* = 15**	**Conestat alfa group *N* = 5**	*P*-value*
**Demographics**			
Age in years	59 (51–71)	60 (54–81)	0.5
Male sex, *N* (%)	12 (80)	4 (80)	1.0
Obesity, *N* (%)	7 (47)	2 (40)	1.0
Arterial hypertension, *N* (%)	7 (57)	4 (80)	0.3
Diabetes mellitus, *N* (%)	2 (13)	1 (20)	1.0
Chronic lung disease, *N* (%)	3 (20)	1 (20)	1.0
Congestive heart failure, *N* (%)	2 (13)	1 (20)	1.0
Chronic renal failure, *N* (%)	3 (20)	2 (40)	0.6
Cardiovascular disease, *N* (%)	4 (27)	2 (40)	0.6
Charlson Comorbidity index	2 (1–5)	4 (1–4)	0.7
**Findings on presentation**			
Symptom duration in days	7 (1–9)	7 (5–8)	0.7
Fever, *N* (%)	13 (87)	2 (40)	0.07
Cough, *N* (%)	11 (73)	4 (80)	1.0
Diarrhea	4 (27)	3 (60)	0.3
Dyspnea	5 (33)	2 (40)	1.0
NEWS2 score	5 (3–8)	7 (5–8)	0.5
Lung involvement in %^a^	28 (18–36)	24 (14–36)	0.7
Lymphocytes, × 10^9^/l	0.7 (0.3–1.04)	1.1 (0.5–1.3)	0.3
CRP, mg/l	85 (69–166)	72 (28–230)	0.7
LDH, U/l	425 (319–506)	354 (238–433)	0.5
D-dimer, μg/ml^b^	1.1 (0.5–2.3)	1.2 (0.7–4.2)	0.4
Ferritin day, μg/L^b^	1817 (1,165–2,677)	906 (501–1,280)	0.1
Creatinine, μmol/L	91 (70–131)	106 (72–215)	0.7
**Treatment**			
Lopinavir/ritonavir, *N* (%)	13 (87)	5 (100)	1.0
Hydroxychloroquine, *N* (%)	14 (93)	5 (100)	1.0
Tocilizumab, *N* (%)	6 (40)	2 (40)	1.0
Antibiotic treatment, *N* (%)	9 (60)	4 (80)	0.6
**Outcome**			
Length of stay in days^c^	10 (8–13)	10 (7–22)	0.9
Intubation, *N* (%)	6 (40)	1 (20)	0.6
Death, *N* (%)	4 (27)	0 (0)	0.5
Intubation or death, *N* (%)	8 (53)	1 (20)	0.3

*CRP, C-reactive protein; LDH, lactate dehydrogenase*.

*Numerical Data are Presented as Median and Interquartile Range, Categorical Data as Frequency and Percentage*.

a *Lung involvement was determined from computed tomography scans of the chest*.

*Reference values of laboratory parameters: CRP (normal range <10 mg/L), LDH (normal range 135–225 U/L for males and 135–214 U/L for females), D-dimer (normal range 0.19–0.5 μg/ml), lymphocytes (normal range 0.9–3.3 x 10^9^/l), ferritin (normal range 30–300 μg/l for males and 10–200 μg/l for females), creatinine (normal range 49–97 μmol/L for males and 42–80 μmol/L for females)*.

b *For controls, measurements up to day 3 were considered; still, D-dimer and ferritin concentrations were not available in 4 and 5 control patients, respectively*.

c *only survivors were counted*.

* *The Fisher's exact test was used for comparisons of categorical variables and the Mann-Whitney U-test to compare continuous variables in the control group of patients vs. the group of patients treated with conestat alfa. All testing was two-tailed*.

### Course of Laboratory Parameters

Inflammatory markers decreased or stabilized in 4/5 patients (Figure 1B). The individual course of inflammatory markers, which were measured inconsistently in the control population is depicted in Figure S1. C1INH concentrations were elevated in all patients before conestat alfa treatment (range, 0.45–0.71 g/L, normal range 0.21–0.39, for individual values see Table 1). Concentration of plasma complement activation products C4d and C5a decreased within 5 days in most patients (Figure 1C). SARS-CoV-2 viral loads in nasopharyngeal swabs declined in 4/5 patients.

### Safety Events After Administration of Conestat Alfa

Patient 2 developed acute on chronic renal failure on day 2 attributed to severe diarrhea secondary to COVID-19 infection and LPV/r treatment. After hydration with Ringer's lactate and sodium bicarbonate solution and cessation of LPV/r, renal function returned to almost baseline before discharge. Grade 2 liver injury (alanine transaminase elevation >2.5–5.0x upper limit of normal) was documented in 3 patients, which was attributed to treatment with LPV/r, amoxicillin/clavulanic acid, tocilizumab or paracetamol. Incident asymptomatic deep venous thrombosis was diagnosed on routine ultrasound in one patient (patient 4) after admission to the ICU, and the patient was anticoagulated. Clot resolution was documented on a subsequent ultrasound before ICU discharge.

## Discussion

A range of strategies have been proposed and are being evaluated to dampen hyper-inflammation in COVID-19 such as corticosteroid therapies, antibodies against IL-1 or IL-6, treatments interfering with the interferon pathway and anti-tumor necrosis factor therapies (28). In particular, the CS has gained attention as a potential therapeutic target in COVID-19 patients (29, 30), and first experiences targeting C3 and C5 of the complement system have been published (31, 32). However, in this case series, we investigated – to the best of our knowledge – for the first time a strategy of interfering with 3 plasmatic cascades including the CS (i.e., CS, CAS, and KKS) in 5 non-critically ill patients with severe COVID-19. Treatment with conestat alfa over 48 h was well-tolerated. No signal of impaired viral clearance emerged from our case series.

The clinical condition improved in all but 1 patient, reflected by immediate defervescence, improvement in oxygen requirements, and a decline in systemic inflammation and markers of complement activation. Notably, 4/5 patients treated early did not require mechanical ventilation despite the presence of risk factors such as markedly elevated inflammatory proteins. In the majority of patients (4/5) IL-6 levels were markedly elevated (>80 ng/ml) during the disease course, a finding shown to predict respiratory failure and mechanical ventilation, previously (33).

Similar to previous studies in sepsis (20), C1INH protein levels were elevated in COVID-19 patients consistent with an acute phase response. However, elevated C1INH levels may not be sufficient to block ongoing extensive CS, CAS, and KKS activation in COVID-19. An increased amount of modified (cleaved) inactive C1INH in patients with severe sepsis was documented previously, which may indicate a relative C1INH-deficient state (34). Of note, concentration of complement activation products C4d and C5a decreased following conestat alfa administration. Early supplementation of C1INH with conestat alfa is thus a plausible treatment option in selected patients with COVID-19, thereby inhibiting upstream complement proteases and its associated over-activation of the CS in COVID-19 (13, 29).

### Limitations

The present study has a number of limitations. First, this was a small case series with a heterogeneous patient population and no controls. Patients may have improved without conestat alfa treatment, although the immediate defervesence and decrease in inflammatory markers is reassuring. Second, sustained complement inhibition may not have been achieved with this current regimen, given the short half-life of conestat alfa and limited treatment duration. Third, all patients were treated with multiple other agents used off-label for the treatment of COVID-19 including LPV/r and hydroxychloroquine.

## Conclusion

In this uncontrolled case series of 5 non-critically ill patients with severe COVID-19 pneumonia, administration of conestat alfa over 48 h to inhibit the CS, CAS and KKS was well-tolerated and associated with improvement in the clinical condition of 4 patients. Consequently, we have initiated a randomized controlled trial to investigate this promising approach (ClinicalTrials.gov, number NCT04414631).

## Data Availability Statement

The raw data supporting the conclusions of this article will be made available by the authors, without undue reservation.

## Ethics Statement

The studies involving human participants were reviewed and approved by Ethics Committee of Northwest and Central Switzerland (EKNZ 2020-1013). The patients/participants provided their written informed consent to participate in this study.

## Author Contributions

SM, BG, MT, and MO designed the study. MO, PU, SM, PC, IH, MR, PS, GS, MT, and SB performed the study, collected, analyzed, and interpreted the data. PU and MO drafted the manuscript. All authors critically revised the manuscript.

## Conflict of Interest

BG reports being employed by Pharming Group NV. MT reports receiving grants from the Swiss National Science Foundation, Roche, Novartis, and Idorsia outside of the submitted work. MO reports receiving consulting fees from Pharming Biotechnologies B.V. during the conduct of the study and grants from Pharming Biotechnologies B.V. outside the submitted work. The remaining authors declare that the research was conducted in the absence of any commercial or financial relationships that could be construed as a potential conflict of interest.

## Supplementary Material

Figure S1 Temporal changes of body temperature, plasma C-reactive protein levels, lymphocyte counts and lactate dehydrogenase levels in 15 control patients. For patient 9, no C-reactive protein and lactate dehydrogenase levels are available during the admission period. Day 0 denotes the day of admission.
