# Supplementary figures and images for: Treatment of COVID-19 With Conestat Alfa, a Regulator of the Complement, Contact Activation and Kallikrein-Kinin System
Source: Front Immunol. 2020 Aug 14;11:2072. doi: 10.3389/fimmu.2020.02072 (PMC7456998; doi:10.3389/fimmu.2020.02072)

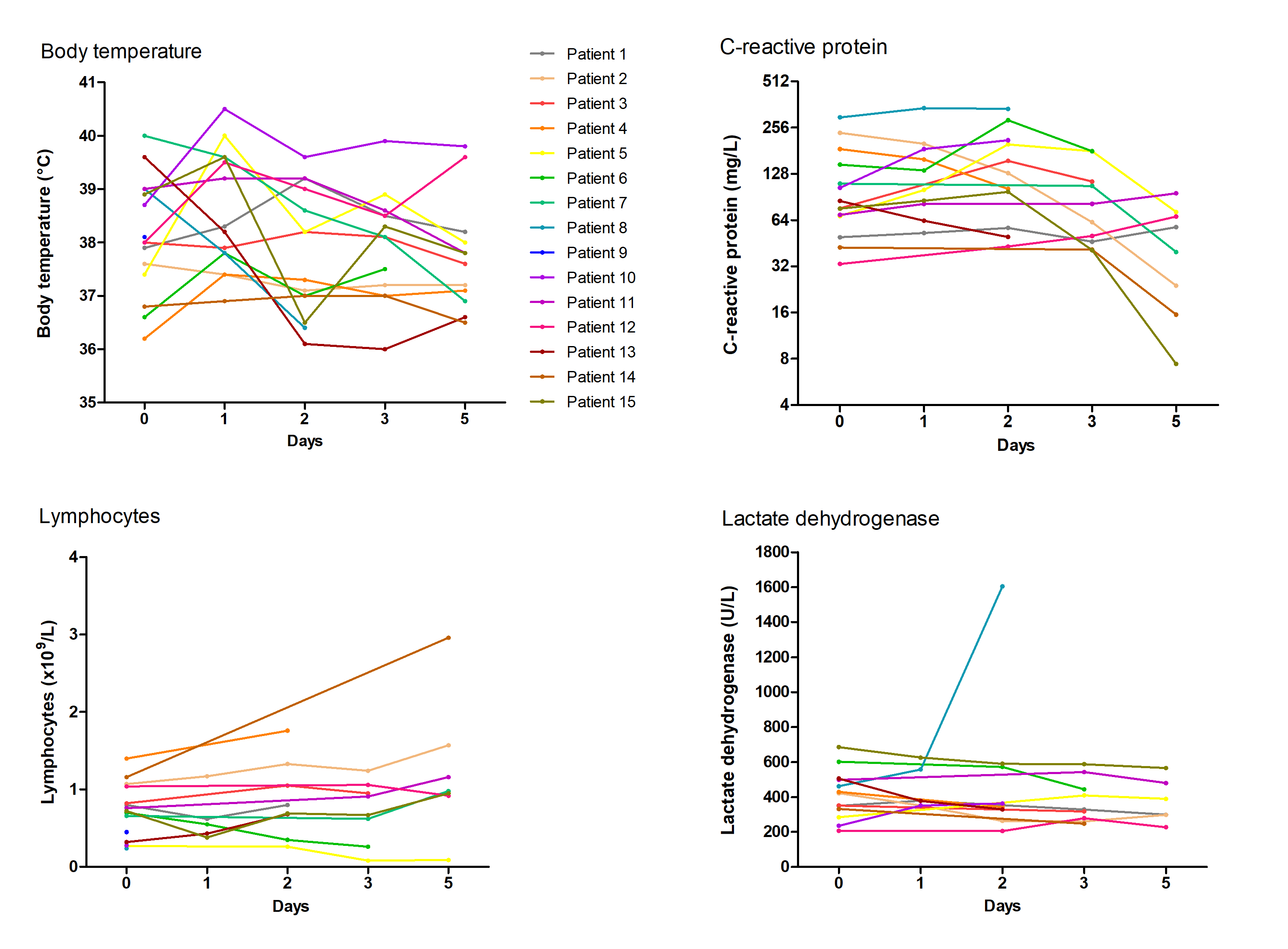

Supplement: Figure S1 — Temporal changes of body temperature, plasma C-reactive protein levels, lymphocyte counts and lactate dehydrogenase levels in 15 control patients. For patient 9, no C-reactive protein and lactate dehydrogenase levels are available during the admission period. Day 0 denotes the day of admission. [file Image_1.TIF]
